# Unique features of apicoplast DNA gyrases from *Toxoplasma gondii* and *Plasmodium falciparum*

**DOI:** 10.1186/s12859-014-0416-9

**Published:** 2014-12-19

**Authors:** Soshichiro Nagano, Ting-Yu Lin, Jyotheeswara Reddy Edula, Jonathan Gardiner Heddle

**Affiliations:** Heddle Initiative Research Unit, RIKEN, 2-1 Hirosawa, Wako, Saitama 351-0198 Japan; Current address: Department of Molecular Protozoology, Research Institute for Microbial Diseases (RIMD), Osaka University, 3-1 Yamadaoka, Suita, Osaka 565-0871 Japan

**Keywords:** Topoisomerase, DNA gyrase, malaria toxoplasmosis, Negative supercoiling, Apicoplast

## Abstract

**Background:**

DNA gyrase, an enzyme once thought to be unique to bacteria, is also found in some eukaryotic plastids including the apicoplast of Apicomplexa such as *Plasmodium falciparum* and *Toxoplasma gondii* which are important disease-causing organisms. DNA gyrase is an excellent target for antibacterial drugs, yet such antibacterials seem ineffective against Apicomplexa. Characterisation of the apicoplast gyrases would be a useful step towards understanding why this should be so. While purification of active apicoplast gyrase has proved impossible to date, *in silico* analyses have allowed us to discover differences in the apicoplast proteins. The resulting predicted structural and functional differences will be a first step towards development of apicoplast-gyrase specific inhibitors.

**Results:**

We have carried out sequence analysis and structural predictions of the enzymes from the two species and find that *P. falciparum* gyrase lacks a GyrA box, but *T. gondii* may retain one. All proteins contained signal/transport peptides for localization to the apicoplast but *T. gondii* Gyrase B protein lacks the expected hydrophobic region. The most significant difference is in the GyrA C-terminal domain: While the cores of the proteins, including DNA binding and cleavage regions are essentially unchanged, both apicoplast gyrase A proteins have C-terminal domains that are significantly larger than bacterial counterparts and are predicted to have different structures.

**Conclusion:**

The apicoplast gyrases differ significantly from bacterial gyrases while retaining similar core domains. *T. gondii* Gyrase B may have an unusual or inefficient mechanism of localisation to the apicoplast. *P.falciparum* gyrase, lacks a GyrA box and is therefore likely to be inefficient in DNA supercoiling. The C-terminal domains of both apicoplast Gyrase A proteins diverge significantly from the bacterial proteins. We predict that an additional structural element is present in the C-terminal domain of both apicoplast Gyrase A proteins, including the possibility of a β-pinwheel with a non-canonical number of blades. These differences undoubtedly will affect the DNA supercoiling mechanism and have perhaps evolved to compensate for the lack of Topoisomerase IV in the apicoplast. These data will be useful first step towards further characterisation and development of inhibitors for apicoplast gyrases.

**Electronic supplementary material:**

The online version of this article (doi:10.1186/s12859-014-0416-9) contains supplementary material, which is available to authorized users.

## Background

Apicomplexa are a group of unicellular protist parasites, most of which contain a plastid known as the apicoplast. Apicoplasts are the result of secondary endosymbiosis in which a prokaryotic cyanobacterium was incorporated into a unicellular eukaryote which was subsequently engulfed by a second eukaryote [1,2]. As a result of this process, the apicoplast contains the remnants of the bacterial plasmid and has four membranes, which from outside inward are the second host’s endomembrane, the first host’s plasma membrane and the two apicoplast membranes [2].

The Apicomplexa are of interest because they count amongst their number several important human pathogens. Foremost amongst these are *Plasmodium* species, responsible for malaria, a disease which in 2010 infected approximately 200 million people resulting in over 600,000 deaths [3] and *Toxoplasma gondii* which can cause dangerous complications in the immune-compromised, is classified by the CDC as a “neglected parasitic disease” and is the biggest cause of death from foodborne illness in the USA [4].

Treatments for both toxoplasmosis (caused by *T. gondii*) and malaria exist but are not without their drawbacks. Most notably in the case of malaria, resistance to existing treatments is widespread [5] and has recently come to include artemisinin, the major component of the most effective current treatment [6,7]. Progress has been made in development of a malaria vaccine but results of large scale clinical trials have been disappointing [8] although most recent small scale trials do show promise [9]. Drug resistance in *T. gondii* is also regarded as problematic [10].

Apicoplasts are known to be essential for the survival of apicomplexan cells due to their numerous roles (reviewed by van Dooren and Striepen [2]) these include synthesis of heme, iron-sulfur clusters, fatty acids and isoprenoids. The requirement for an apicoplast was initially demonstrated in *T. gondii,* which was unable to survive when apicoplast DNA replication was inhibited [11], or when the apicoplast was absent [12]. In *Plasmodium* their essential function in the blood stages appears to be synthesis of isoprenoid precursors [13].

The indispensability of the apicoplast together with fact that it is a eubacteria-derived plastid raises the possibility of exploiting it for specific targeting of pathogenic Apicomplexa with antibacterial drugs without affecting the human host [14].

**Figure 1:**
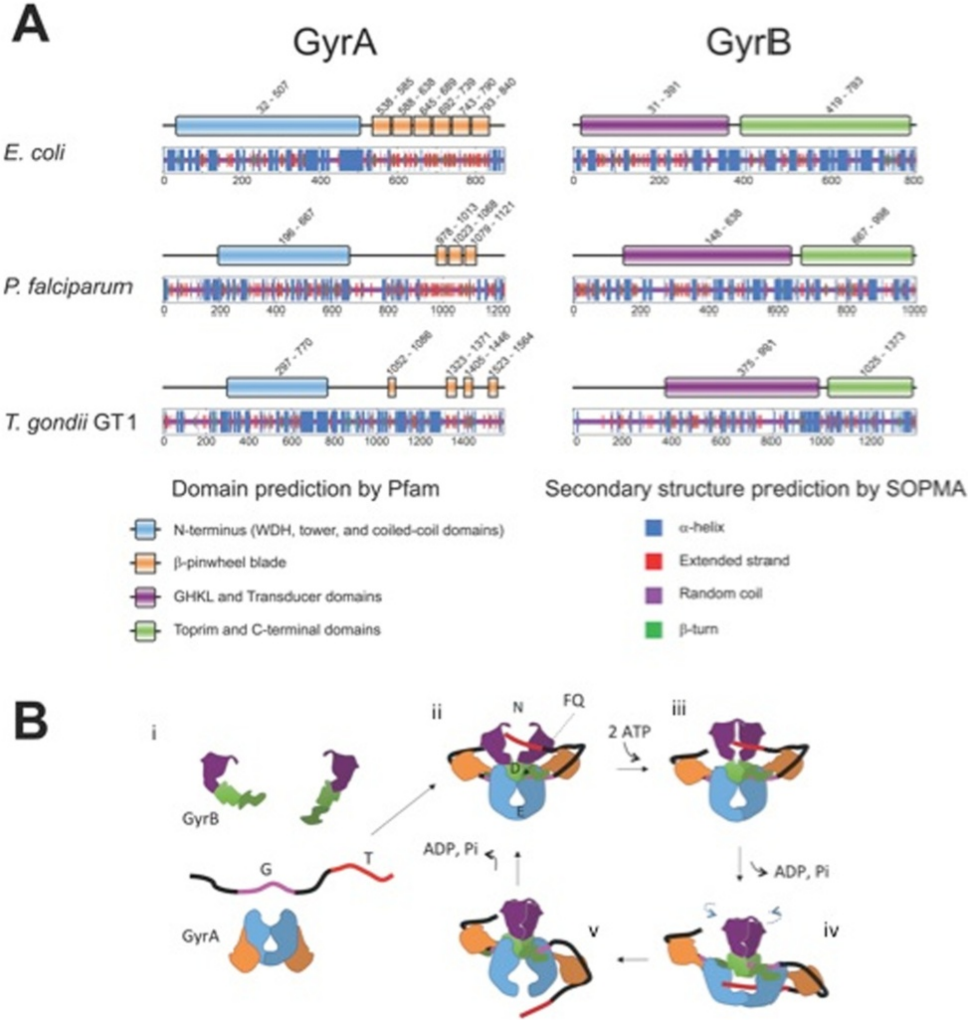
**Comparison of gyrase proteins and mechanism of enzyme action.** **(A)** Domains and secondary structures predicted from amino acid sequences of GyrA and GyrB from *E. coli*, *P. falciparum*, and *T. gondii* GT1. Proteins are depicted at constant length regardless of their actual molecular weight. Domains were predicted by Pfam [20] and secondary structures were predicted by the SOPMA server [21]. **(B)** Schematic mechanism of supercoiling by DNA gyrase: (i) GyrB and GyrA dimers assemble on a piece of DNA. (ii) A protein-DNA complex is formed when the G-segment (“G”) binds to the active site of the enzyme at the DNA gate (“D”). (iii) The N-terminal regions of GyrB (shown in purple, “N”) dimerize upon ATP binding and capture the T-segment (“T”). The G segment is cleaved across both strands. This cleaved conformation is trapped by gyrase poisons such as the fluoroquinolones (FQ) and is lethal to the cell. (iv) The DNA gate is opened and the T-segment is transported into the lower cavity of the enzyme. (v) The exit gate (“E”) of the enzyme opens and the T-segment passes out. A single cycle of supercoiling is complete while two ATP are hydrolyzed.

One of the most successful antibacterial drug targets to date has been DNA gyrase (“gyrase”), a type II topoisomerase that has the unique ability to carry out ATP-dependent negative supercoiling of DNA [15]. Gyrase consists of GyrA and GyrB proteins with the functional enzyme being an A_2_B_2_ heterotetramer. The supercoiling mechanism [16] is complex and includes a step in which the enzyme produces a transient double-stranded break in the substrate DNA (see Figure 1). GyrA consists of an N-terminal domain which contains a DNA binding region and the active site tyrosine, involved in DNA cleavage via formation of a phosphotyrosine bond and a C-terminal domain (CTD) that wraps DNA with the appropriate handedness for negative supercoil generation and delivers it to GyrB. The CTD structure responsible for this wrapping is termed a β-pinwheel fold [17]. This is similar to the β-propeller fold [18] overall but with different topology in its repeating units (“blades”). GyrB consists of an N-terminal domain containing the region responsible for ATP binding and hydrolysis and the transducer region which connects the ATPase domain to the TOPRIM (topoisomerase-primase [19]) domain. The C-terminal region of GyrB contains the TOPRIM domain involved in the DNA cleavage reaction (Figure 1).

Gyrase poisons work by inhibiting the religation of cleaved DNA, leading to fragmentation and cell death. The highly successful fluoroquinolone class of antibacterials function in this way and also target topoisomerase IV (“topo IV”), a closely related bacterial type II topoisomerase, in the same fashion. Topo IV itself is structurally highly similar to DNA gyrase, also functioning as a heterotetramer (ParC_2_, ParE_2_). Like gyrase it requires ATP hydrolysis to carry out its function, which is mainly decatenation of daughter chromosomes rather than negative supercoiling. Differences in the C-terminal domain of GyrA/ParC subunits appear to account for this difference in function [22].

Until recently, gyrase was thought to occur only in bacterial cells. It is now known that, while apparently not occurring in humans and most other higher eukaryotes, it is found in plant plastids [23]. A malarial gyrase, whose existence had long been suggested by evidence such as the activity of fluoroquinolones against *Plasmodium* and *T. gondii* [11,24-27], was finally shown to exist through sequencing of the *P. falciparum* genome [28] and is localized to the apicoplast. Apicoplast gyrase has been mooted as a potentially useful therapeutic target [14,29] although fluoroquinolone activity *in vitro* culture is usually much higher than in an infected host where results are variable. In the case of *in vitro* experiments with *T. gondii* for example, ciprofloxacin (CFX) has an IC_50_ of 27.9 μg/ml [30] and in the case of *P. falciparum* in *in vitro* culture, initial results suggested an IC_50_ for CFX of 1.7 μg/ml for 24-hour experiments [26]. Mahmoudi *et al*. carried out a comprehensive *in vitro* test of 25 quinolones and fluoroquinolones against blood stages of *P. falciparum* [31] and found an IC_50_ for CFX of approximately 9.2 μg/ml and 3.4 μg/ml against chloroquine sensitive and resistant strains respectively. These numbers compare to IC_50_s for CFX against *E. coli* in the approximate range of 0.011-0.015 μg/ml [32].

Interestingly, the way in which CFX affects *T. gondii* appears different to the effect on *P. falciparum* as *T. gondii* shows a clear “delayed death” where extending treatment time lowers the IC_50_, something which appears not to occur in *P. falciparum* for CFX [33] although it is seen for some other molecules.

There is much less information regarding the effectiveness of these drugs against the pathogens in an infected host (“*in vivo*”). CFX, while active against *T. gondii* in *in vitro* culture, apparently shows little effect against the parasite *in vivo* [27]. In contrast, the fluoroquinolone trovafloxacin is unusual in that it does show *in vivo* activity [27]. Poor *in vivo* performance in general could be related to complications caused by parasite life cycle or simply the additional physical barriers in place in the host cell. Differences could also be due to differences in structure or mechanism of action of the enzymes themselves*.* Mature *T. gondii* gyrase (Tg-gyrase) is 142% the size of *E. coli* gyrase (Ec-gyrase) in terms of number of amino acids and *P. falciparum* gyrase (Pf-gyrase) is 116%, giving much scope for potential deviation from the *E. coli* “norm”. To understand the poor effectiveness of gyrase-poisoning antibacterials against Apicomplexa it is very important to biochemically and biophysically characterize purified Pf- and Tg-gyrases *in vitro*.

Despite problems in expression and purification of Pf-gyrase proteins, some biochemical experimental work has been carried out utilising various domains (summarised in Table 1): Full length Pf-GyrB has been characterized for ATPase activity which was found to be DNA-stimulated [34,35] and shows a *K*_*cat*_ similar to that reported for the 43 kDa domain of Ec-GyrB [34,36]. An N-terminal region of Pf-GyrB (residues 121–497, equivalent to the Ec-GyrB 43 kDa ATPase domain) has also been produced and characterized and shows ATP hydrolysis activity at a somewhat lower rate than its *E. coli* counterpart [35].

**Table 1:** **Summary of experiments previously carried out with** ***P. falciparum*** **gyrase proteins**

**Pf protein**	**Partner protein**	**Test**	**Result**	**References**
GyrB (various constructs)		ATPase	Decreased ATP turnover rate compared to *E. coli* protein ATPase activity can be enhance	Dar et al., 2007 [35]; Ram et al., 2007 [34]; Dar et al., 2009 [37]
		DNA binding	DNA binding activity through 45 amino acid insert region	Dar et al., 2009 [37]
		Conformational change in response to nucleotide	Clamp closure can be modulated by nucleotide binding	Dar et al., 2009 [37]
		Dimerization	Dimerization occurred in the absence of ATP	Dar et al., 2007 [35]
GyrB	Ec-GyrA (full length)	ATPase	Enhanced ATP turnover rate	Dar et al., 2007 [35]
		Supercoiling	Lower supercoiling activity compared to E. coli gyrase	Dar et al., 2007 [35]; Dar et al., 2009 [37]
		DNA cleavage	Introduced DNA breaks	Dar et al., 2007 [35]; Dar et al., 2009 [37]
GyrB	Pf-GyrA NTD	DNA cleavage	Introduced DNA breaks	Dar et al., 2007 [35]
		Supercoiling	Failed in DNA supercoiling	Dar et al., 2007 [35]
GyrA NTD	Ec-GyrB (full length)	DNA cleavage	Introduced DNA breaks	Dar et al., 2007 [35]

Pf-GyrB also shows ATP-hydrolysis and DNA supercoiling activities when complemented with Ec-GyrA [35]. Furthermore the enzyme appears resistant to drugs that function by disrupting the ATP-binding site with the Ki value of coumermycin reported to be 500 times that of the *E. coli* protein [35] and the Ki value of novobiocin being approximately 3.5 times higher for Pf-GyrB compared to Ec-GyrB [34].

While full-length Pf-GyrA (residues 156–1222) has not been successfully expressed and purified, it has proved possible to produce the N-terminal DNA cleavage-reunion domain (residues 163–540) [35]. This domain shows CaCl_2_-induced and CFX-induced DNA cleavage when combined with Pf-GyrB [35]. The expression and characterization of the full-length CTD of Pf-GyrA has not been reported to date, although an N-terminal portion of it (residues 723–887) has been produced [35].

The lack of a purified full length Pf-GyrA has undoubtedly hampered progress in understanding Pf-gyrase. Still less is known of the Tg-gyrase proteins, the purification of which has not been reported to date (as is the case for all other apicomplexan gyrases). If these enzymes could be purified and characterized biochemically and structurally in comparison with standard eubacterial gyrase the data generated could help in the design of new inhibitors and may also point to a better understanding of the mechanism of action of gyrase itself.

In terms of structure, alignments for *P. falciparum* have suggested a close match between the 43 kDa N-terminal GyrB domain of Ec-gyrase containing the ATP-binding and hydrolysis site and the equivalent region of Pf-GyrB and between the 59 kDa N-terminal cleavage-reunion domain of Ec-GyrA and equivalent region of Pf-GyrA [34]. However, to the best of our knowledge such alignments have not been carried out for the C-terminal regions of Pf-GyrA and GyrB which is where the majority of sequence divergence is located. Tg-gyrase proteins appear never to have been subject to structural prediction studies.

Until purified holoenzymes become available, *in silico* approaches may be useful. Here we have used sequence analysis and structural alignments/predictions to compare DNA gyrase from *P. falciparum* and *T. gondii* to known bacterial enzymes (Table 2). Ultimate verification will of course require biochemical experiments but we hope that these initial results will spur further investigations into these interesting gyrases.

**Table 2:** **Summary of the protein sequences used in this study**

**GyrA**			
**Organism**	**Protein length**	**Molecular weight (Da)**	**Accession number**
*E. coli*	875	96977.3	[GenBank: ACI81094]
*M. tuberculosis*	838	92345.1	[GenBank: AFR90340]
*S. aureus*	889	99624.9	[GenBank: NP_373244]
*S. enterica*	878	97020.9	[GenBank: WP_001281283]
*X. campestris*	899	99027.3	[GenBank: WP_010366603]
*P. cynomolgi*	1266	141638.6	[GenBank: P_004225009]
*P. falciparum*	1222	143144.1	[GenBank: AAN36310]
*P. knowlesi*	1173	133858.5	[GenBank: XP_002262302]
*P. vivax*	1263	144004.5	[GenBank: EDL47594]
*T. gondii* GT1	1594	175836.3	[GenBank: EPR58555]
*T. gondii* ME49	1278	142457.1	[GenBank: XP_002369962]
*E. coli* (ParC)	752	83831.0	[GenBank: NP_289596]
**GyrB**			
**Organism**	**Protein length**	**Molecular weight (Da)**	**Accession number**
*E. coli*	804	89866.6	[GenBank: BAA20341]
*M. tuberculosis*	686	75323.9	[GenBank: NP_334414]
*S. aureus*	644	72523.7	[GenBank: NP_373243 ]
*S. enterica*	826	92159.9	[GenBank: WP_006542968]
*X. campestris*	814	89688.5	[GenBank: WP_010376402]
*P. cynomolgi*	908	102112.0	[GenBank: XP_004225170]
*P. falciparum*	1006	116106.2	[GenBank: AAN36469]
*P. knowlesi*	992	112346.0	[GenBank: XP_002262476]
*P. vivax*	936	104905.8	[GenBank: EDL44204]
*T. gondii* GT1	1386	148239.4	[GenBank: EPR58339]
*T. gondii* ME49	1257	134533.3	[GenBank: XP_002371198 ]
*E. coli* (ParE)	630	70243.7	[GenBank: NP_417502]

Accession numbers were retrieved from NCBI (http://www.ncbi.nlm.nih.gov/).

We show that the apicomplexan gyrases, as well as the obvious differences in size, contain signal and translocation peptides, which differ according to protein and species. Pf-gyrases contain numerous asparagines including repeat sequences. Perhaps the most significant differences between the proteins are found at the GyrA CTD, which varies significantly in size compared to the bacterial counterparts from which they show considerable divergence. We speculate that this divergence may include a DNA wrapping β-pinwheel domain with a different size/number of blades than has been seen to date in all known gyrases and/or an additional domain of unknown function. These differences may be related to modulation of supercoiling control in apicoplasts, which are not known to contain topoisomerase IV.

## Results and discussion

### Sequence comparisons

#### Sequence analyses of GyrA

Bioinformatics analyses were carried out in order to gain deeper insights into the unique properties of Pf- and Tg-gyrases. Sequence alignments for Tg- and Pf- GyrAs and GyrBs all aligned best with topo IV structures. This likely reflects the lack of structural data for the relevant regions of gyrase proteins (i.e. full length GyrB and GyrA including the C-terminal DNA wrapping domains) rather than an indication that the structures actually more closely resemble *E. coli* topo IV.

Amino acid sequences of diverse gyrases were analysed using the MEGA analysis software [38]. Examples of several bacterial GyrAs and GyrBs were compared to examples from *Plasmodium* species along with sequences from *T. gondii* and *E. coli* topoisomerase IV. Two analyses were carried out. Firstly pairwise distances between sequences were calculated (Additional file 1: Tables S1 and S2). These showed that for gyrases, the differences in amino acid sequences were greatest between the bacterial gyrases and the *Plasmodium* gyrases for both GyrA and GyrB. Topoisomerase IV, as would be expected, was also the most different in the case of ParE compared to GyrBs, but in fact less different than the *Plasmodium* GyrA proteins in the case of ParC. Tg-GyrA fell between bacterial and *Plasmodium* sequences in terms of difference and Tg-GyrB was similar to *Plasmodium* proteins. The maximum parsimony bootstrap consensus trees for the proteins show a similar result with two clear clusters, one for bacterial proteins and another for *Plasmodium* proteins with topoisomerase IV and *T. gondii* gyrases being somewhat variable outliers (See Additional file 1: Figure S1). Sequences were also aligned with ClustalW 2.1 [39,40] which showed that 25.6% of residues were identical between Ec-GyrA and Pf-GyrA. The value changes when analyses are delimited to individual domains identified by the Pfam server. Percentages of identical residues for the N-terminal domain (Ec-GyrA 32*–*507 encompassing WHD, tower, and coiled-coil domains) and the C-terminal domain (Ec-GyrA 538–840 encompassing the β-pinwheel domain) were 34.1% and 18.0%, respectively. These observations are rationalized by lower homology of the C-terminal domain of Pf-GyrA with respect to Ec-GyrA. Analysis of the amino acid sequences of Ec-GyrA using the Pfam domain prediction server results in six β-sheet blades of the β-pinwheel motif being predicted in accordance with reported experimental results [41]. In contrast, only three such motifs are predicted from the Pf-GyrA sequence with the E-value set to 10, the maximum value. This prediction is consistent with secondary structures predicted by the SOPMA structure prediction server [21], where β-sheets are predicted for those regions of Pf-GyrA (Figure 1A). At face value, this suggests the presence of a small β-pinwheel domain consisting of a cluster of no more than three blades (Figure 1, Additional file 1: Figure S2). All known gyrases have 6 blades [42]. Given that individual blades in Pf-GyrA appear to be approximately the same size as those found in other gyrases, it is difficult to envisage three blades alone to wrap DNA with a sufficiently acute angle to deliver it to the DNA clamp. Two possible explanations are that *i)* Pf-GyrA may be an example of a topo II with fewer than six β-pinwheel motifs (as is seen for *E. coli* topo IV [42]). *ii)* Pf-GyrA does in fact contain a β-pinwheel with a larger number of blades but they are of a sequence highly divergent from known structures and are thus not recognised by Pfam. For Tg-GyrA, the percentage of identical residues to Ec-GyrA is 33.5%. In terms of the distribution of identical residues, similarity can be observed to that seen between Ec-GyrA and Pf-GyrA, i.e. a higher identity is found in the N-terminal domain (41.5%) compared to the C-terminal domain (26.7%).

The N-terminal 59 kDa fragment of Ec-GyrA bears a domain similar to the DNA-binding domain of the catabolite-activator protein (CAP) containing a helix-turn-helix (HTH) motif and the tower domain [43]. A hallmark of topoisomerase, the active site tyrosine that forms a transient phosphodiester bond with the end of the G-segment DNA is found in the HTH motif, which is part of the positively charged groove. A cluster of conserved residues comprises the active site of Ec-GyrA based on the crystal structure of the 59 kDa fragment. Those residues (Ec-GyrA Arg32, Lys42, Arg46, Arg47, Arg121, Tyr122) are perfectly conserved among Pf- and Tg- GyrAs, consistent with their role and confirming that the cleavage-reunion domain likely functions in an identical manner to that of other gyrases.

Next, the “GyrA-box” and its vicinity were considered. The GyrA-box is a positively charged motif (Sequence Q++GG + G, where + is any positively charged residue [42]) found in blade 1 of the β-pinwheel of the GyrA CTD and is known to be necessary for DNA supercoiling and wrapping [44,45]. GyrA from species such as *E. coli* have a conserved proline at position 636 (Ec-GyrA numbering) close to the hinge between blades 1 and 2 and this was thought to introduce a tilt in the packing between blades, causing the pinwheel to be out of plane, giving positive handedness to the DNA wrap [46]. Deletion of this proline in Ec-GyrA leads to a 2-3-fold decrease in supercoiling activity [46]. In some other species lacking the proline, such as *B. burdorferi*, the CTD is planar [17]. It is also planar in the case of 6-bladed Topo IV CTDs [47]. However, in *M. tuberculosis* gyrase, there is no conserved proline and yet the CTD is tilted [48]. These results suggest that other mechanisms in addition to/instead of the “conserved” proline are responsible for the non-planar shape.

**Figure 2:**
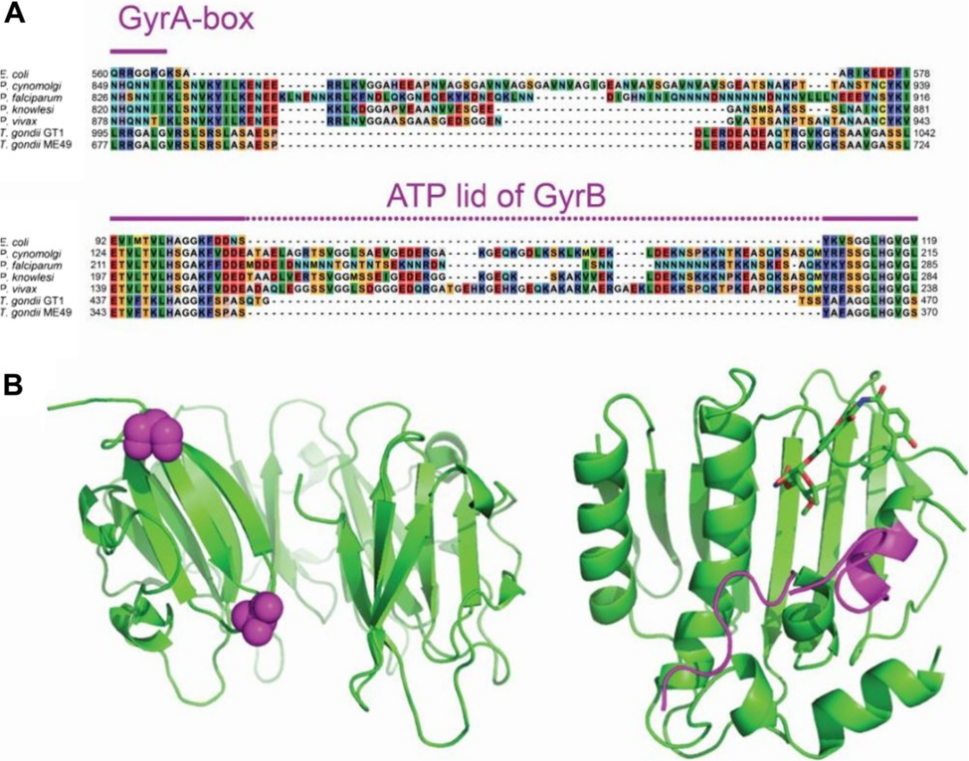
**Locations of asparagine repeats in** ***P. falciparum*** **gyrase genes and the structures of** ***E. coli*** **gyrases containing the corresponding regions. (A)** The asparagine-repeat of Pf-GyrA is located in a region close to the region corresponding to the GyrA box of prokaryotic gyrases. This region often appears disordered in crystal structures. Two residues (Ser555 and Asp576) flanking the disordered region containing the GyrA box (560-QRRGGKG-566) are highlighted by magenta spheres (PDB code 1ZI0 [41] chain A). **(B)** The asparagine-repeat is encompassed in the *Plasmodium* specific insertion of gyrase B. The region corresponds to the ATP lid of Ec-GyrB, highlighted by magenta on the 24 kDa fragment structure (PDB code 1AJ6 [49]).

Our alignments (Figure 2, Additional file 1: Figure S2) show that Pf-GyrA lacks a GyrA-box while a potential GyrA-box is present in Tg-GyrA (as a 996-RRGALGV-1006 motif similar to the canonical sequence found in *E. coli*). It is notable that this Tg-GyrA box does not coincide with blade sequences as predicted by Pfam (Additional file 1: Figure S2) although it does coincide with the location of a β-pinwheel as predicted by I-TASSER when the sequence of Tg-GyrA CTD is submitted (see below). Interestingly, the sequence in place of the GyrA-box in Pf-GyrA has a negative charge (Pf-GyrA Asn821-Ser913 pI: 5.50). This stands out among other *Plasmodium* sequences in this region, which have pIs ranging between 7.19 and 8.17. The loop region containing the GyrA-box in Ec-GyrA (Ser555-Asp576) has a high pI of 8.16. The corresponding region of Tg-GyrA (Ser990-Ala1039) is similarly positively charged with its pI at 8.69, which is in contrast to that of Pf-GyrA (Asn821–Tyr914, pI: 5.38). Pf*-*GyrA and Tg-GyrA both have apicoplast specific insertions in the region C-terminal to the GyrA-box (71 and 30 additional residues compared to Ec-GyrA, respectively). The moderately conserved proline (Ec-GyrA Pro636), is not present in either *Plasmodium* nor *Toxoplasma* gyrases. As a consequence of the lack of the GyrA box in Pf-gyrase we may expect that it is unable to efficiently supercoil DNA (as has been found for other gyrases lacking a GyrA box [44]). In the case of Tg-GyrA, four β-pinwheel blades predicted by Pfam are spread throughout the C-terminal region rather than being clustered together. In contrast, the homology model based on the mature amino acid sequence of Tg-GyrA by I-TASSER suggests that the majority of the β-pinwheel blades are located N-terminal to those predicted by Pfam.

#### Sequence analyses of GyrB

The overall percentage of identical residues between Ec*-*GyrB and Pf-GyrB was 38.5%. Unlike the case of Ec-GyrA and Pf-GyrA, the identity is spread more homogenously throughout the length of the proteins. The percentage of identical residues at the N-terminal domain (*E. coli* 31–391) is 35.7%, whereas for the C-terminal domain (*E. coli* 419–793) it is 40.7%. The percentage of identical residues between Ec*-*GyrB and Tg-GyrB is 35.1% (N-terminal domain: 35.6%, C-terminal domain: 32.9%).

Interestingly, Pf-GyrB includes an insertion of 45 amino acids in its TOPRIM domain which is essential for activity [37]. This same insert is present in all *Plasmodium* species but is absent from the other 9 GyrB genes we considered. Our alignments also confirmed a 49-amino acid insert in part of the ATPase domain corresponding to the ATP lid of Ec-GyrB (Figure 2). We find that this insert, which has been noted previously [34] is unique to *Plasmodium* GyrBs amongst the proteins we investigated.

#### Size comparison

**Figure 3:**
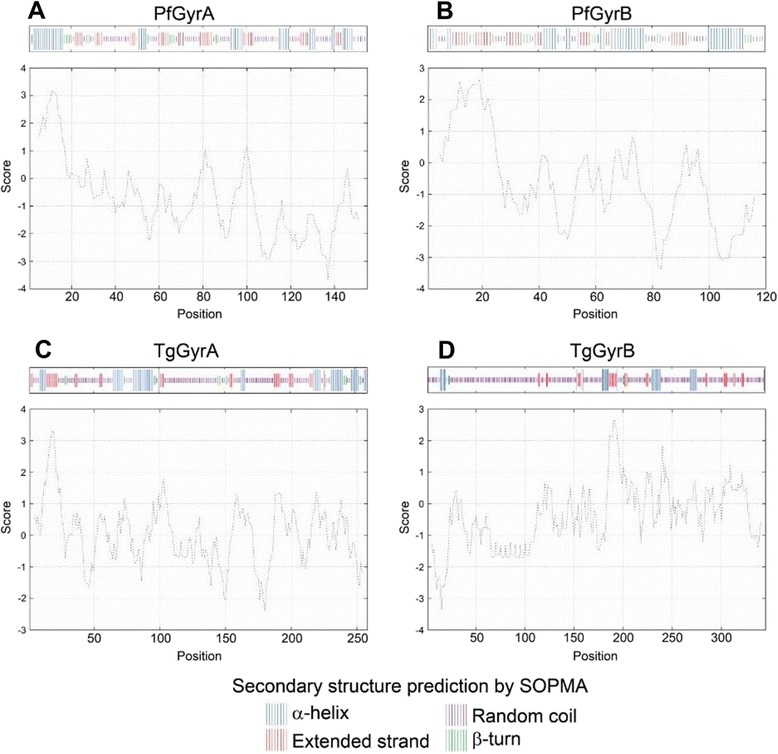
**Analyses of the signal/transit peptides of apicomplexan gyrase sequences.** Secondary structure prediction and hydrophobicity plot of residues 1–155 of Pf-GyrA **(A)** and residues 1–120 of Pf-GyrB **(B)**. Both sequences feature hydrophobic α-helices in the N-terminus. Secondary structure prediction and hydrophobicity plot of residues 1–259 of Tg-GyrA **(C)** and residues 1–261 of Tg-GyrB **(D)**. On the contrary to other proteins, Tg-GyrB does not feature a hydrophobic region in its N-terminus.

The most striking difference between the proteins in comparison to their *E. coli* counterparts is in overall size. GyrA and GyrB in *E. coli* are 875 and 804 amino acids in length respectively; in Pf-gyrase the lengths of mature proteins are 1067 and 886 amino acids respectively while in Tg-gyrase they are 1336 and 1041 amino acids respectively. The “extra” sequences in the apicomplexan gyrases are not entirely devoid of secondary structure as predicted by SOPMA [21] (Figure 1A, Figure 3). Those additional structural elements could confer unique properties to apicoplast gyrases, for example a 45 amino acid insertion in the TOPRIM domain of Pf-GyrB known to play a role in ATPase activity, DNA binding, and DNA cleavage [37]. There are cases, however, where the non-homologous regions are predicted to form low complexity regions (LCRs), which appear correlated with single residue repeats. The *P. falciparum* proteome has previously been reported to be rich in asparagine repeats [50,51]. The feature is partially attributed to its AT-rich genome, but asparagine repeats are not found in other species of equally AT-rich *Plasmodium spp.* [51]. Asparagine repeats are thought to render the proteome susceptible to forming intracellular aggregates, but given the presence of a potent Hsp110 and other chaperones, they may provide a positive selective pressure by playing a role in immune evasion [52,53]. The proportion of asparagine residues in the coding sequences of gyrase A and B were compared between four species in the *Plasmodium* genus: *P. falciparum*, *P. vivax*, *P. cinomolgi*, and *P. knowlesi* (Table 3). It is clear that the gyrase genes of *P. falciparum* encode the highest proportion of asparagine in accordance with Muralidharan and Goldberg [51]. Additionally, not only do Pf-gyrase proteins feature a quantitatively higher proportion of asparagine residues, but they are qualitatively unique because Pf-GyrA and Pf-GyrB each have regions where asparagines are clustered into repeats (Additional file 1: Figure S3). For Pf-GyrB this region (253–261) is located in the area corresponding to the ATP-lid of the GHKL Bergerat fold of the ATPase domain [54], whereas in Pf-GyrA the repeat (888–902) is located in the region that corresponds to the GyrA box of the C-terminal 33 kDa fragments in prokaryotic GyrA. In both cases the repeats are located in areas corresponding to loop regions in the predicted structures (Figure 2).

**Table 3:** **Proportions of asparagine residues in the coding sequences of gyrases A and B in four species of** ***Plasmodium***

	***P. falciparum***	***P. vivax***	***P. cynomolgi***	***P. knowlesi***
Gyrase A	14.65 (10.97)	7.44	7.98	9.04
Gyrase B	13.72 (10.83)	10.15	8.04	7.56

Figures are given in percentages (%). The figures in parentheses for *P. falciparum* are proportions of asparagine residues in the signal peptides (Pf-GyrA: 1–155, Pf-GyrB: 1–120).

In contrast to Pf-gyrases, Tg-gyrases are rich in serines (Table 4). Unlike the asparagine repeats in Pf-gyrases, these serine repeats are primarily concentrated in the N-terminal signal peptide regions (Additional file 1: Figure S4). Additionally, Tg-GyrB has a serine repeat in a region, which equates to the transducer domain of *E. coli* GyrB 43 kDa fragment.

**Table 4:** **Proportion of serine resides in gyrases** ***of T. gondii*** **GT1 with respect to the full-length sequence and the signal peptide**

	**Full length protein**	**Signal peptide**
GyrA	1 – 1594, 9.91%	1 – 258, 23.26%
GyrB	1 – 1386, 20.06%	1 – 345, 40.58%

Signal peptide *of T. gondii* GT1 gyrases were inferred from aligned sequences (Additional file 1: Figure S5). Residue numbers are shown, followed by the proportion of serine residues as apercentage.

We also compared the signal and transit peptides of the proteins. A typical apicoplast-targeting protein contains an N-terminal signal peptide followed by the apicoplast transit peptide for protein import into the apicoplast [55]. The amino acid sequence varies in transit peptides from different apicomplexans, for example, lysine is common in *P. falciparum* while arginine is common in *T. gondii* [56,57]*.* Those basic residues near the N-terminus of the transit peptide are reported to be important for faithful transport into the apicoplast and an algorithm has been established to identify the signal and transit peptides in multiple Apicomplexa species [58]. We analyzed N-terminal regions of *Plasmodium* and *Toxoplasma* gyrases that are expected to play key roles in translocation. Submission of *Plasmodium* and *Toxoplasma* gyrase sequences to the SOPMA [21] and Protscale [59] servers results in identification of a short hydrophobic α-helix predicted for each *Plasmodium* gyrase gene (Figure 3). This is consistent with previous work showing that the signal peptides of apicoplast-targeted proteins in *P. falciparum* comprise a hydrophobic region [60]. In contrast, analyses of the Tg-GyrB sequence indicate different characteristics at the N-terminal region (Figure 3): Surprisingly for a protein that is predicted to be translocated to the apicoplast, the N-terminal signal peptide of Tg-GyrB is markedly non-hydrophobic which is in contrast to Tg-GyrA, Pf-GyrA and Pf-GyrB. This observation could imply an inefficient direction of Tg-GyrB across the endoplasmic reticulum in the first step of the secretory pathway.

### Structure prediction and alignments

#### GyrA

**Figure 4:**
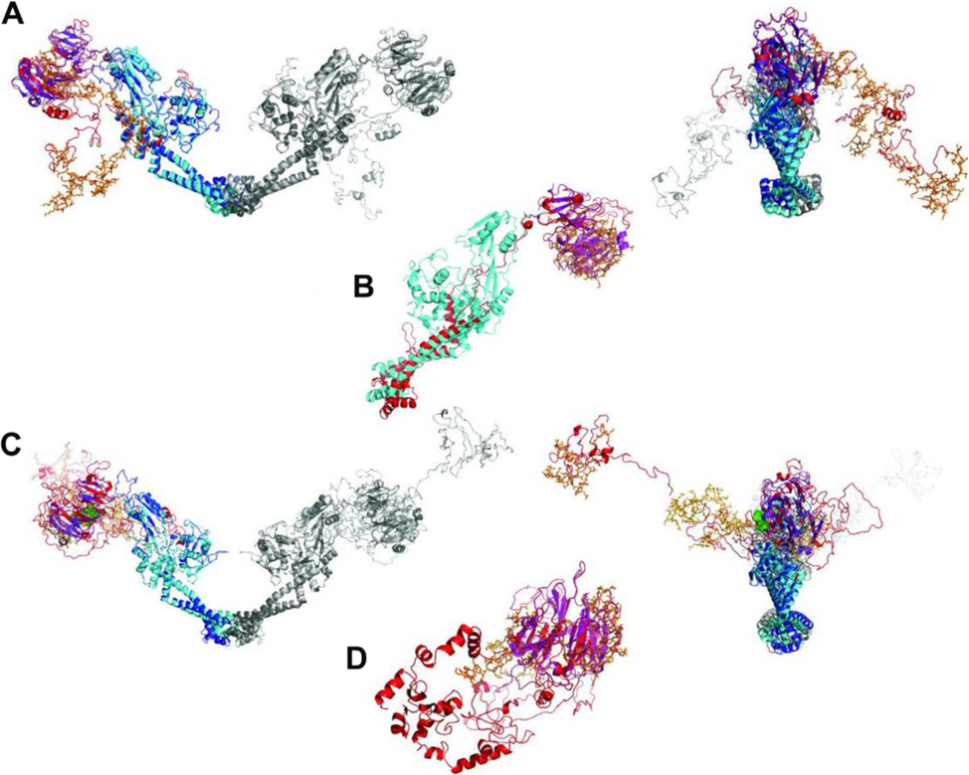
**Homology models of Pf-GyrA and Tg-GyrA by I-TASSER.** **(A)** Alignment of Pf-GyrA with Ec-ParC (1ZVU [21]). One monomer of ParC is dark grey, the other is cyan for ParC with the β-pinwheel (497-740) colored purple. One monomer of Pf-GyrA is light grey and the other colored according to the results of the Pfam prediction as follows: N-terminal residues 196-667 are in dark blue, and the predicted β-pinwheel blades (538-840) are orange and in stick representation. The remaining sequences, which are not predicted to have any secondary structure, are red. Right hand side is the same alignment as A but rotated 90° around the y axis. **(B)** Alignment of the CTD (671-1222) of Pf-GyrA with Ec-ParC (1ZVU). Part of the model aligns with the a-helical bundle of the template, but is likely due to a-helices being predicted in that region of Pf-GyrA, rather than specific homology to the region in the template. **(C)** Alignment of Tg-GyrA with Ec-ParC. One monomer of ParC is dark grey, the other monomer is cyan for ParC with the β-pinwheel (497-740) colored purple. One monomer of Pf-GyrA is light grey and the other is colored according to the results of the Pfam prediction as follows: N-terminal residues 297-770 are dark blue, and the predicted β-pinwheel blades (1052-1086, 1323-1371, 1405-1446 and 1523-1564) are orange and in stick representation. The likely GyrA-box of Tg-GyrA is shown as green spheres. The remaining sequences that are not predicted to have any secondary structure are red. Right hand side is the same alignment as C but rotated 90° around the y axis. **(D)** Alignment of the CTD (775-1594) of Tg-GyrA with the template (3L6V). As for Pf-GyrA, a-helices are also predicted N-terminal to the b-pinwheel motifs.

Homology modelling of full-length Pf-GyrA (minus the signal and transit peptide sequences) was carried out using I-TASSER [61,62]. The protein was predicted to align well with a number of known GyrA/ParC structures (see Additional file 1: Table S3) with the structure of full-length ParC from *E. coli* (1ZVU [42]) ranking top. Figure 4A shows the alignment between the predicted Pf-GyrA structure and ParC. The rmsd was 1.37 Å. The alignment shows that the N-terminus of Pf-GyrA approximately equivalent to the 59 kDa region of *E. coli* GyrA aligns well with *E. coli* ParC, including the DNA breakage-reunion site, the coiled-coil region and the C-gate.

Pf-GyrA CTD structure prediction by I-TASSER differs from Pfam results in that residues identified by Pfam as part of the β-pinwheels in the CTD (residues 978–1121, shown in orange in Figure 4A) do not align with the equivalent structure in ParC but residues 710–968 do align with the 5-bladed ParC pinwheel. Given that the two predicted regions do not overlap, it may be that the actual structure consists of a large β-pinwheel consisting of both regions combined resulting in eight blades. This would be unusual as all gyrase β-pinwheels known have six blades. The equivalent region of topo IV in various organisms is more variable and includes both 3-bladed and 8-bladed pinwheels [42].

This inconsistency is attributable to a constraint to the structural homology calculations that derives from the fact that the ParC template structure features the pinwheel domain immediately C-terminal to the N-terminal domain. In order to create a homology model of the C-terminal domain of Pf-GyrA without such constraint, the sequence of the C-terminal region (671–1222) was submitted to I-TASSER on its own. This results in the pinwheel domain being predicted in a region different from when the fuller sequence is submitted (Figure 4B). Homology models based on the five best templates consistently features the β-pinwheel domains beginning in residues 875–877 and ending in residues 1220–1222 which covers all blades predicted by Pfam, and some blades predicted by I-TASSER using the mature sequence, to give a total of 6 blades. Also, the five best models consistently feature α-helices between residues 698–848. This is qualitatively similar to the prediction made by Dar *et al*. [35] in predicting a coiled-coil between the NTD and the CTD of GyrA, and hence legitimises the prediction of an additional structural element in *Plasmodium* GyrA compared to bacterial counterparts.

When the full length Tg-GyrA sequence (omitting the signal and transit sequences) was submitted to I-TASSER, the results as shown in Figure 4C were obtained. As with Pf-GyrA, the protein aligned best with the same *E. coli* ParC structure (pdb 1ZVU [42]). The rmsd was 1.91 Å. As was the case for the Pf-GyrA protein, there is good alignment with the cleavage reunion and N-terminal portion of ParC. In addition, of the residues identified as β-pinwheel blades by Pfam; (1052–1086, 1323–1371, 1405–1446 and 1523–1564) only the most N-terminal (1052–1086) overlaps with the β-pinwheel blades of ParC, the others being dispersed throughout the C-terminus in regions not aligned with any part of the ParC structure. Interestingly, the region predicted to be the most N-terminal blade is not recognized as any domain/motif by Pfam. The I-TASSER alignment places unaligned, largely unstructured sequences both N and C-terminal to the β-pinwheel. The C-terminal 500 residues beginning at residue A1096 of Tg-GyrA were not assigned as corresponding to any existing structure in ParC by I-TASSER. Similarly to Pf-GyrA, the sequence of the C-terminal region of Tg-GyrA (775–1594) was submitted to I-TASSER on its own (Figure 4D). The models based on the top four templates consistently predict α-helices in residue range of 788–945, followed by β-pinwheel motifs between 974–1505. The latter range only covers three out of four blades predicted by Pfam but, as for the CTD of Pf-GyrA, a total of 6 β-pinwheel blades is predicted in total. Clearly, the details of precisely which parts of the C-terminus are predicted to be a pinwheel depends on the software employed and the submitted amino acid sequence. Prediction of α-helices N-terminal to the β-pinwheel domain is similar to the case in Pf-GyrA, and suggests the presence of α-helices between the NTD and the CTD as being a common element among apicomplexan GyrAs.

Homology models obtained from I-TASSER reveal little evidence of gross structural deviation by Pf-GyrA and Tg-GyrA at the catalytic core and its surroundings. This is unsurprising given that the sequence identity is higher for the N-terminal domains in both GyrA and GyrB as described above. The most effective gyrase targeting antibacterials, the fluoroquinolone gyrase poisons, bind to a site made primarily of residues of the cleavage-reunion domains in GyrA and bound DNA [15]. The absolute conservation of amino acid sequence of this region in Pf and Tg-GyrA means that the binding site for gyrase poisons is likely unchanged. In Tg-GyrA, the exact start residue of the mature protein is not fully established, thus the possibility of a relatively large structural element at the N-terminus is not completely ruled out.

#### GyrB

**Figure 5:**
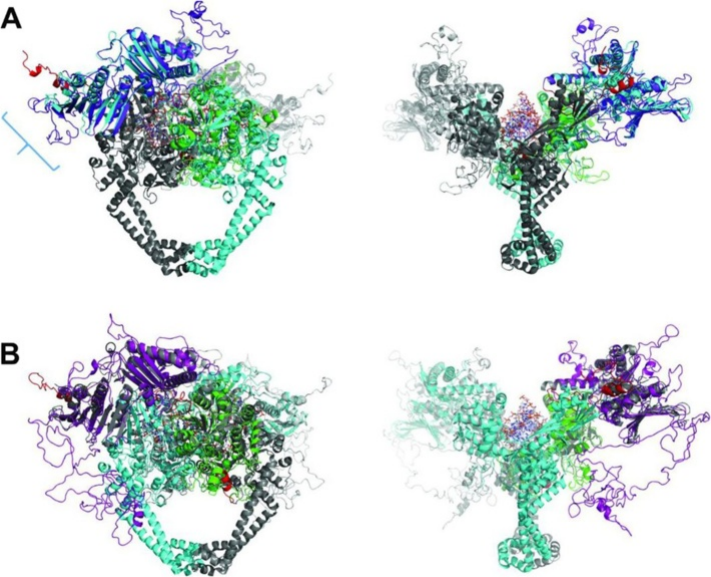
**Homology models of Pf-GyrB and Tg-GyrB by I-TASSER. (A)** Alignment of Pf-GyrB with topoisomerase IV complexed with DNA (pdb 4I3YH [63]). One monomer of the topo IV dimer is shown in dark grey, the other in cyan. One monomer of the Pf-GyrB is colored light grey, the other is colored as follows (numbering includes the signal and translocation peptide) residues 148–340 (roughly equivalent to GHKL domain), purple, residues 704–875 (roughly equivalent to the transducer domain, green) residues 910–1195 (roughly equivalent to TOPRIM and C-terminal tail), yellow. Unassigned sequence is shown in red. DNA bound to the DNA cleavage region is shown in stick format. Other DNA is removed for clarity. Bracketed loop is a *Plasmodium* specific insert. **(B)** Alignment of Tg-GyrB with Topoisomerase IV complex with DNA (pdb 4I3YH [63]). Coloring is as follows: Residues 291–876 (roughly equivalent to GHKL and transducer domains), purple, residues 910–1258 (roughly equivalent to TOPRIM and C-terminal tail), yellow. Unassigned sequence is shown in red. DNA bound to the DNA cleavage region is shown in stick format. Other DNA is removed for clarity. For each presentation, an alternative view rotated horizontally by 90° is shown on right hand side.

GyrB from *P. falciparum* lacking the signal and transit peptides was submitted to I-TASSER. The structure of full-length bacterial GyrB is not known and the alignment returned was best matched to a *S. pneumoniae* topo IV structure 4I3H [63] consisting of full-length ParE (equivalent to GyrB) fused to a 55 kDa region of ParC (equivalent to GyrA). The rmsd was 1.63 Å. The results (Figure 5A) shows that there is good structural homology overall with only a small number of continuous non-homologous regions. One interesting example is a region in the vicinity of the unique 49 amino acid insert (Pf-GyrB 224–272) previously identified in the ATP lid [34] (Figure 5A). Previous alignments suggested the 49 amino acid insert to be flanked by regions with high homology to other known GyrB sequences and form an unstructured loop [34]. On the contrary, the homology modelling by I-TASSER predicts the residues 225–320 to form a loop region (highlighted in Figure 5A). This inconsistency is likely an instance of “loop swapping” (see Methods section).

Tg-GyrB was also submitted to I-TASSER and like Pf-GyrB aligns well with the same *S. pneumoniae* topo IV structure 4I3H [63] with an rmsd of 1.14 Å. Due to its extra length, Tg-GyrB contains a greater number of longer, unassigned loops. These loop regions are clustered around the GHKL region (Figure 5B). Overall, in both Pf- and Tg-GyrBs core domains seem to be largely preserved and this includes the regions involved in DNA binding (Figure 5).

**Figure 6:**
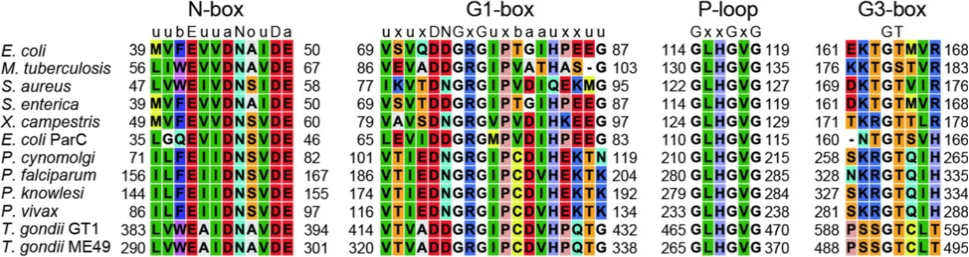
**Conserved motifs found within the ATPase domain of gyrases and** ***E. coli*** **topo IV.** High degree of conservation can be observed, although some divergence is also present.

GyrB contains the ATPase domain and it is clear from the aligned sequences of these domains that many key motifs are conserved between bacterial and apicoplast gyrases (Figure 6). Motifs conserved among GHKL fold termed N (uubEuuaNouDa), G1 (uxuxDNGxGuxbaauxxuu), G2 (uGxxGxouxSxxxuoxbuTuxT), and G3-box (Tx_n_GT) can be identified (u, conserved bulky hydrophobic residues; b, basic residues; a, acidic residues) [54]. Indeed the ATPase activities of Pf-GyrB have been demonstrated [35,37,64]. The sequence of the GyrB subunit especially the ATP binding pocket is highly conserved among bacterial and apicoplast gyrases. For example, the residues located in a structural pocket, including N46, E50, R76, I78, K103, V118, and T165 known for ATP binding and ATPase inhibitors binding in Ec-GyrB [65] are conserved in Pf-GyrB. Two residues involved in the quinolone-binding pocket (also called quinolone resistance-determining region, QRDR [66,67]), N426 and K447 of Ec-GyrB are also conserved in Pf-GyrB. The similarity in drug interacting residues may explain the observation that known gyrase-targeting antibacterials drugs can show similar effects toward Pf-gyrase in ATPase activity and DNA cleavage activity assays [34,35]. A significant divergence is seen in Pf-GyrB where an insertion is present in the “lid” region covering the ATP binding site [34] (see below). In Tg-GyrB the clustering of continuous non-assigned sequences around the GHKL domain hints that this region is most structurally divergent and may even point to the presence of an additional domain. ATP binding and/or hydrolysis may also be affected in Pf-GyrB due to the insert in the ATP lid, possibly explaining the decrease in hydrolysis rate compared to Ec-GyrB [35]. Indeed it has previously been speculated that the presence of unstructured loop regions in the ATPase domain may affect ATPase activity and the effectiveness of inhibitors, which bind in that region [34]. The extra sequence in this region could perhaps also provide scope for binding of new inhibitors.

## Conclusions

In summary, we have carried out a comprehensive sequence and structural comparison of two apicomplexan gyrases to standard bacterial (*E. coli*) gyrase and have found a number of differences which may have both relevance to enzyme function and consequences in terms of effectiveness of therapeutics as well as providing useful data for future structural studies.

While the results presented in this work are not definitive there are sufficient data to enable some tentative speculation, which should be considered in the light of recent findings from *M. tuberculosis*: This organism also has a gyrase but lacks topo IV. *M. tuberculosis* gyrase has a greater decatenation ability relative to negative supercoiling ability compared to Ec-gyrase [68,69], something which is thought to be due to decreased DNA-stimulated ATPase activity and a truncated “tail” in the CTD which combine to inhibit the enzyme from achieving as high a level of DNA duplex under-winding as the *E. coli* enzyme [48]. Further research has shown that this gyrase has a calcium binding site and that that gyrase in *M. tuberculosis* can shift between gyrase-like and topo IV-like activities via the modulatory action of Ca^2+^ which binds in the linker region between the NTD and CTD of GyrA [70]. This is proposed to alter the position of the β-pinwheels relative to the remainder of the enzyme such that supercoiling may be favored in the absence of Ca^2+^ while disfavored in its presence, allowing relaxation/decantation to occur. The fact that the greatest variation in Pf- and Tg-gyrases compared to Ec-gyrase is in the CTD strongly suggests that this is due to a role in affecting or modulating the balance between gyrase-like and topo IV-like activities. Tg-gyrase, in contrast to Pf-gyrase, has a likely GyrA box but lacks an insert in the ATP lid meaning that these mooted activity modifiers are not available to it. In this instance, compensation for the lack of topo IV is presumably achieved at least in part through its unusually large GyrA CTD, which may affect extent and/or position of the DNA wrap. It is also the case that additional structural elements found in *Plasmodium* and *Toxoplasma* gyrases may be responsible for modulating their activity by means not found in prokaryotes. These may pose an additional target for drugs in the future. For example, we may envisage that inhibiting the apicoplast targeting of gyrases will be lethal to Apicomplexa.

Further biochemical studies on individual gyrase proteins from these organisms as well as holoenzymes in conjunction with high-resolution studies will be required to answer outstanding questions and assess their suitability as targets for development of therapeutics.

## Methods

### Sequence comparisons

Amino acid sequences of proteins were aligned with ClustalW 2.1 using the default parameters [39]. The percentages of sequence identities were calculated by dividing the number of identical residues by the total aligned sequence length without insertions. Aligned sequences were viewed using CLC Sequence Viewer (CLC bio). Aligned amino acid sequences of gyrase and topoisomerase IV proteins from various species were inputted into MEGA (v. 6.06 beta) [38]. Pairwise distances between sequences were computed (the number of amino acid differences divided by the total number of amino acids compared). Gaps and missing data were deleted and no variance estimation method was used. A maximum parsimony tree was calculated using the Jones-Taylor-Thornton model assuming uniform rates among sites. Phylogeny was tested using the bootstrap method with 500 bootstrap replications.

### Structure prediction

Structure predictions were carried out using I-TASSER [61,62]. In all cases, amino acid sequences for the proteins, lacking the predicted signal and transit sequences were submitted to the I-TASSER server. Default settings were used with no additional restraints employed. From the top 10 identified structural analogues returned the best alignment was chosen based on TM-score ranking. The top ranking alignment templates were *E. coli* ParC [63] for Pf-GyrA, Pf-GyrA CTD (residues 671–1222) and Tg-GyrA, *S. pneumoniae* ParC-ParE55 fusion protein [63] for Pf-GyrB and Tg-GyrB and *X. campestris* GyrA CTD for Tg-GyrA CTD (residues 775–1594). PDB coordinates were visualised using PyMOL [71]. All of the proteins considered were significantly longer than the homologous regions in the model templates. This has an important consequence for the homology models generated. When additional sequences are present then clearly not all sequences can be aligned to a template. In some cases we observe that where sequences flanking these loop regions themselves have poor similarity to the template, then “loop swapping” may occur where the flanking sequence is in fact designated a loop and the loop is assigned structure. This does occur in parts of our structure but has little effect on the overall model.

### Structural alignments

Dimeric models of homology models of the proteins as generated by I-TASSER, were created by superposing each homology model onto dimeric crystal structures of the template topoisomerases using Coot [72]. The SSM Superpose function was used, and root mean square deviation (RMSD) of the distances between alpha carbons of the aligned residues were calculated. This allowed us to see regions of the Pf or Tg protein, which matched well with a known gyrase protein structure and, more importantly those regions where poor homology was predicted.

### Availability of supporting data

All supporting data associated with this manuscript are included as additional files.

## Additional file

Additional file 1.
**Provides additional data as follows: Additional Tables 1 and 2: Estimates of evolutionary divergence between various GyrA sequences and between various GyrB sequences, Additional Figure 1: Showing molecular phylogenetic analyses for GyrA (and ParC) and GyrB (and ParE). Additional Figure 2: A figure showing sequence alignment of predicted b-pinwheel blades of GyrA.** Additional Figure 3: A figure showing the location of asparagine residues in Pf-gyrase proteins. Additional Figure 4: A figure showing the location of serine residues in Tg-gyrase proteins. Additional Figure 5: Sequence alignments used to infer the starting position of the mature gyrase proteins of *T. gondii* GT1.
