# Supplementary material for: Unique features of apicoplast DNA gyrases from Toxoplasma gondii and Plasmodium falciparum
Source: BMC Bioinformatics. 2014 Dec 19;15(1):416. doi: 10.1186/s12859-014-0416-9 (PMC4297366; doi:10.1186/s12859-014-0416-9)
Supplement: Additional file 1. — Provides additional data as follows: Additional Tables 1 and 2: Estimates of evolutionary divergence between various GyrA sequences and between various GyrB sequences, Additional Figure 1: Showing molecular phylogenetic analyses for GyrA (and ParC) and GyrB (and ParE). Additional Figure 2: A figure showing sequence alignment of predicted b-pinwheel blades of GyrA. Additional Figure 3: A figure showing the location of asparagine residues in Pf-gyrase proteins. Additional Figure 4: A figure showing the location of serine residues in Tg-gyrase proteins. Additional Figure 5: Sequence alignments used to infer the starting position of the mature gyrase proteins of T. gondii GT1. [file 12859_2014_416_MOESM1_ESM.pdf]

Unique Features of Apicoplast DNA Gyrases from *Toxoplasma gondii* and *Plasmodium falciparum*

Soshichiro Nagano[1], Ting-Yu Lin[1], Jyotheeswara Reddy [1,2] and Jonathan Gardiner Heddle [1,*]

[1] Heddle Initiative Research Unit, RIKEN, 2-1 Hirosawa, Wako, Saitama, 351-0198, Japan

[2] Current address: Department of Molecular Protozoology, Research Institute for Microbial Diseases (RIMID), Osaka University, 3-1 Yamadaoka, Suita, Osaka 565-0871, Japan

* To whom correspondence should be addressed. Tel: +81 (0)48-467-4598; Fax: +81 (0)48-462-4701; Email: heddle@riken.jp

Additional File 1

Additional Table 1 Estimates of evolutionary divergence between GyrA sequences: The number of amino acid differences per site from between sequences are shown. The analysis involved 11 amino acid sequences. All positions containing gaps and missing data were eliminated. There were a total of 675 positions in the final dataset. Evolutionary analyses were conducted in MEGA6[1].

Additional Table 2: Estimates of evolutionary divergence between GyrB sequences: The number of amino acid differences per site from between sequences are shown. The analysis involved 11 amino acid sequences. All positions containing gaps and missing data were eliminated. There were a total of 549 positions in the final dataset. Evolutionary analyses were conducted in MEGA6[1].

Additional Table 3: The list of top 5 structural analogues in the PDB to the homology models of apicoplast gyrase proteins generated by the I-TASSER, as identified by TM-Align[2]. Ongoing modifications to I-TASSER and the availability of new crystal structures may change these results in the future.

Addtional Table 4. C-scores of the output homology models that were used for structural analyses.

Additional Table 5. List of top 5 threading templates used by I-TASSER in generation of homology models ordered according to their normalized Z-score. All Z-scores shown herein are above 1, a value indicating a good sequence alignment. A template structure may be represented several times each with their Z-score, because various alignments are possible between the amino acid sequences of the template structure and the query protein. In all cases the amino acid sequences of chain A were used.

Additional Figure 1: Molecular phylogenetic analysis by Maximum Likelihood method for **A**: GyrA (and ParC) and **B**: GyrB (and ParE). The evolutionary history was inferred by using the Maximum Likelihood method based on the JTT matrix-based model[3]. The bootstrap consensus tree inferred from 500 replicates[4] is taken to represent the evolutionary history of the taxa analyzed[4]. Branches corresponding to partitions reproduced in less than 50% bootstrap replicates are collapsed. Initial tree(s) for the heuristic search were obtained by applying the Neighbor-Joining method to a matrix of pairwise distances estimated using a JTT model. The analysis involved 11 amino acid sequences. All positions containing gaps and missing data were eliminated. There were a total of 672 positions in the final dataset for GyrA and 549 positions in the final dataset for GyrB. Evolutionary analyses were conducted in MEGA6[1]. The

trees produced are unrooted.

Additional Figure 2: Sequence alignment of predicted β-pinwheel blades of GyrA. Predictions of β-pinwheel blades were carried out using Pfam[5] for GyrAs from various species. Overall sequence identity is low but characteristic shared features include Ser/Thr at approximately the 7th position and a Gly residue at approximately positions 10 and 30 (marked with arrows). The GyrA box is found only in Ec-GyrA blade 1 (labelled), as far as this alignment is concerned.

Additional Figure 3: Asparagine residues in Pf-gyrase proteins. Asparagine residues are highlighted in cyan, and asparagine-repeats (Pf-GyrA: 888–902, Pf-GyrB: 253-261) are underlined.

Additional Figure 4: Serine residues in Tg-gyrase proteins. Serine residues are highlighted in green, and the signal peptide inferred from sequence alignments (*T. gondii* GT1 GyrA: 1-258, *T. gondii* GT1 GyrB: 1-261, Supplementary Figure 5) are underlined.

Supplementary Figure 5: Sequence alignments were used to infer the starting position of the mature gyrase proteins of *T. gondii* GT1 following the cleavage of the signal pepetide. For *P. falciparum* the first residue of the mature gyrase proteins were adopted according to Dar *et al.*[6].

References

1 Tamura, K., Stecher, G., Peterson, D., Filipski, A. & Kumar, S. MEGA6: Molecular Evolutionary Genetics Analysis version 6.0. *Mol. Biol. Evol.* **30**, 2725-2729, doi:10.1093/molbev/mst197 (2013).

2 Zhang, Y. & Skolnick, J. TM-align: a protein structure alignment algorithm based on the TM-score. *Nucl. Acids. Res.* **33**, 2302-2309, doi:10.1093/nar/gki524 (2005).

3 Jones, D. T., Taylor, W. R. & Thornton, J. M. The rapid generation of mutation data matrices from protein sequences. *Comput. Appl. Biosci.* **8**, 275-282 (1992).

4 Felsenstein, J. Confidence limits on phylogenies: An approach using the bootstrap. *Evolution* **39**, 783-791 (1985).

5 Punta, M. *et al.* The Pfam protein families database. *Nucl. Acids Res.* **40**, D290-301, doi:10.1093/nar/gkr1065 (2012).

6 Dar, M. A., Sharma, A., Mondal, N. & Dhar, S. K. Molecular Cloning of Apicoplast-Targeted Plasmodium falciparum DNA Gyrase Genes: Unique Intrinsic ATPase Activity and ATP-Independent Dimerization of PfGyrB Subunit. *Eukaryot. Cell* **6**, 398-412, doi:10.1128/ec.00357-06 (2007).

Additional Table 1

Estimates of Evolutionary Divergence between GyrA Sequences

GyrA

	E. coli	***S. enterica***	***X. campestris***	***S. aureus***	***M. tuberculosis***	***T. gondii* GT1**	***E. coli* ParC**	***P. cynomolgi***	***P. knowlesi***	***P. vivax***
S. enterica	0.067									
X. campestris	0.321	0.330								
S. aureus	0.451	0.452	0.461							
M. tuberculosis	0.518	0.512	0.513	0.491						
***T. gondii* GT1**	0.640	0.649	0.646	0.619	0.647					
***E. coli* ParC**	0.641	0.646	0.644	0.628	0.667	0.689				
P. cynomolgi	0.722	0.729	0.719	0.719	0.731	0.722	0.751			
P. knowlesi	0.725	0.731	0.719	0.716	0.738	0.725	0.757	0.083		
P. vivax	0.720	0.726	0.713	0.710	0.725	0.711	0.747	0.092	0.118	
P. falciparum	0.717	0.719	0.719	0.713	0.737	0.728	0.757	0.259	0.257	0.283

Additional Table 2

Estimates of Evolutionary Divergence between GyrB Sequences

**GyrB**										
	E. coli	***S. enterica***	***X. campestris***	***S. aureus***	***M. tuberculosis***	***P. cynomolgi***	***P. knowlesi***	***P. vivax***	***P. falciparum***	***E. coli* ParE**
S. enterica	0.027									
X. campestris	0.311	0.304								
S. aureus	0.434	0.430	0.439							
M. tuberculosis	0.464	0.461	0.483	0.439						
P. cynomolgi	0.554	0.546	0.559	0.548	0.581					
P. knowlesi	0.556	0.548	0.559	0.543	0.566	0.038				
P. vivax	0.559	0.554	0.561	0.552	0.576	0.047	0.047			
P. falciparum	0.556	0.548	0.556	0.541	0.568	0.146	0.149	0.155		
***E. coli* ParE**	0.588	0.583	0.590	0.601	0.596	0.643	0.645	0.648	0.645	
***T. gondii* GT1**	0.577	0.572	0.583	0.594	0.590	0.546	0.543	0.543	0.541	0.596

Additional Table 3

Input sequence (Residue numbers)	Rank	TM score	Hit PDB	Hit identity
**PfGyrA (156 – 1222)**	1	0.614	1ZVU chain A (3)	*E. coli* full length ParC
	2	0.408	2XCS chain B (4)	*S. aureus* GyrB C-terminus and GyrA N-terminus complexed with an inhibitor and DNA
	3	0.406	4I3H chain A (5)	*S. pneumoniae* full-length ParE-ParC55
	4	0.405	2XKK chain A (6)	*Acinetobacter baumannii* C-terminal ParE and N-terminal ParC fusion in complex with DNA and fluoroquinolone
	5	0.405	2XCO chain A (4)	*S. aureus* GyrB C-terminus and GyrA N-terminus fusion
**PfGyrB (121 – 1006)**	1	0.666	4I3H chain A (5)	*S. pneumoniae* full-length ParE-ParC55
	2	0.377	1EI1 chain A (7)	*E. coli* GyrB 43 kDa dimer, mutant
	3	0.373	1KIJ chain B (8)	*T. thermophiles* GyrB 43 kDa ATPase domain in complex with novobiocin
	4	0.371	4GFH chain A (9)	Topoisomerase II – DNA complex
	5	0.369	3ZKB chain A (10)	*M. tuberculosis* GyrB ATPase domain
**TgGyrA (259 – 1595)**	1	0.478	1ZVU chain A (41)	*E. coli* full length ParC
	2	0.310	2XKK chain A (6)	*Acinetobacter baumannii* C-terminal ParE and N-terminal ParC fusion in complex with DNA and fluoroquinolone
	3	0.310	2XCT chain B (4)	*S. aureus* GyrB C-terminus and GyrA N-terminus fusion
	4	0.308	4I3H chain A (5)	*S. pneumoniae* full-length ParE-ParC55
	5	0.302	3K9F chain A (11)	*S. pneumoniae* C-terminal GyrB and N-terminal GyrA complex
**TgGyrB (346 – 1386)**	1	0.629	4I3H chain A (41)	*S. pneumoniae* full-length ParE-ParC55
	2	0.343	4GFH chain A (9)	Topoisomerase II – DNA complex
	3	0.333	1EI1 chain A (7)	*E. coli* GyrB 43 kDa dimer, mutant
	4	0.326	3ZKB chain A (10)	*M. tuberculosis* GyrB ATPase domain
	5	0.324	1KIJ chain B (8)	*T. thermophiles* GyrB 43 kDa ATPase domain in complex with novobiocin
**PfGyrA (671 – 1222)**	1	0.610	1ZVU chain A (3)	*E. coli* full length ParC
	2	0.481	3L6V chain A (12)	*X. campestris* GyrA C-terminal domain
	3	0.470	3UC1 chain A (13)	*M. tuberculosis* GyrA C-terminal domain
	4	0.459	1ZI0 chain B (14)	*E. coli* GyrA C-terminal domain
	5	0.455	1WP5 chain A (15)	*E. coli* ParC C-terminal domain
**TgGyrA (775 – 1594)**	1	0.378	3L6V chain A (12)	*X. campestris* C-terminal domain
	2	0.348	3G3N chain A (16)	*M. tuberculosis* GyrA C-terminal domain
	3	0.347	1ZI0 chain B (14)	*E. coli* GyrA C-terminal domain
	4	0.343	1WP5 chain A (15)	*E. coli* ParC C-terminal domain
	5	0.315	1VT4 chain L (17)	*D. melanogaster* apoptosome

Additional Table 4

**Input sequence (Residue numbers)**	**C-score of the output homology model**
PfGyrA (156 – 1222)	1.62
PfGyrA (156 – 1222)	1.41
TgGyrA (259 – 1595)	1.18
TgGyrB (346 – 1386)	1.45
PfGyrA (671 – 1222)	1.87
TgGyrA (775 – 1594)	2.34

Additional Table 5

Input sequence (Residue numbers)	Rank	PDB hit	Normalized Z-score
**PfGyrA (156 – 1222)**	1	4BUL	7.80
	2	1ZVU	3.92
	3	1ZVU	3.95
	4	1ZVU	6.99
	5	1ZVU	5.15
**PfGyrB (121 – 1006)**	1	4I3H	3.15
	2	3ZKB	2.93
	3	4I3H	4.35
	4	3ZKB	3.82
	5	1EI1	2.67
**TgGyrA (259 – 1595)**	1	4BUL	8.60
	2	1ZVU	3.69
	3	1ZVU	3.17
	4	1ZVU	6.47
	5	1ZVU	4.95
**TgGyrB (346 – 1386)**	1	4I3H	2.36
	2	3ZKB	2.67
	3	4I3H	3.98
	4	3ZKB	3.66
	5	1EI1	2.81
**PfGyrA (671 – 1222)**	1	3L6V	2.09
	2	1ZVU	5.49
	3	3L6V	2.99
	4	3L6V	4.18
	5	3L6V	3.88
**TgGyrA (775 – 1594)**	1	3L6V	1.53
	2	3L6V	1.68
	3	3L6V	3.13
	4	3L6V	3.13
	5	3L6V	1.73

Additional Figure 1

Molecular Phylogenetic Analysis by Maximum Likelihood method for GyrA, ParC and GyrB

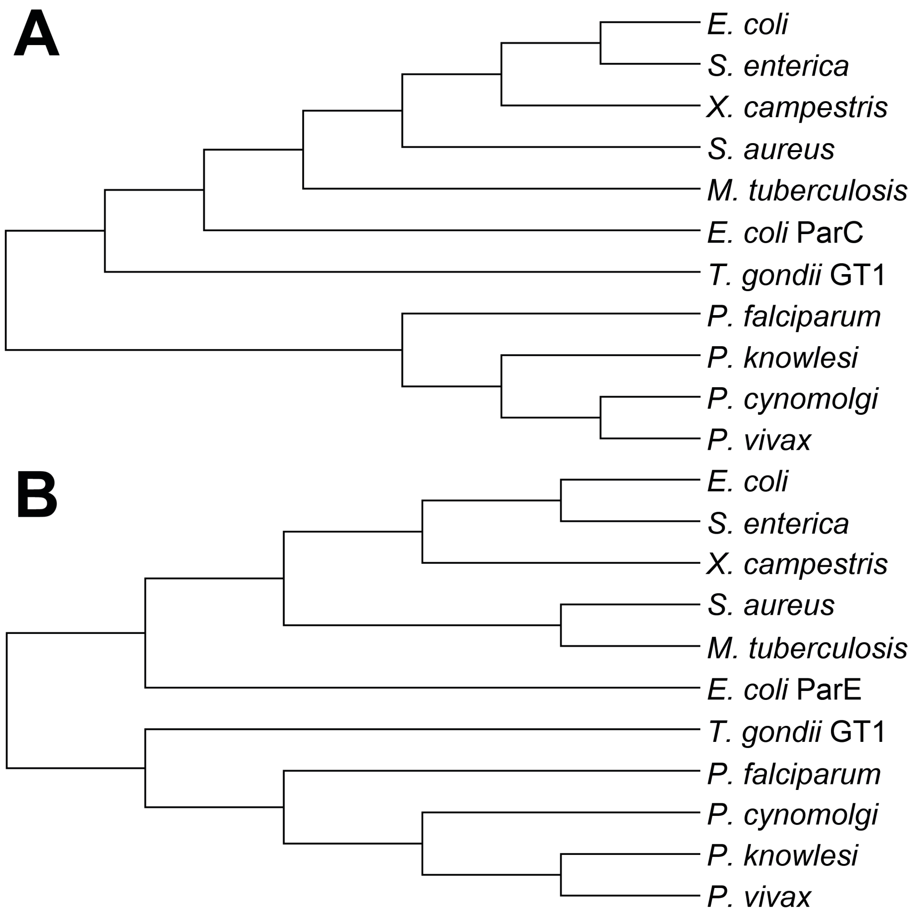

Additional Figure 2

Sequence Alignment of Predicted β-pinwheel Blades of GyrA

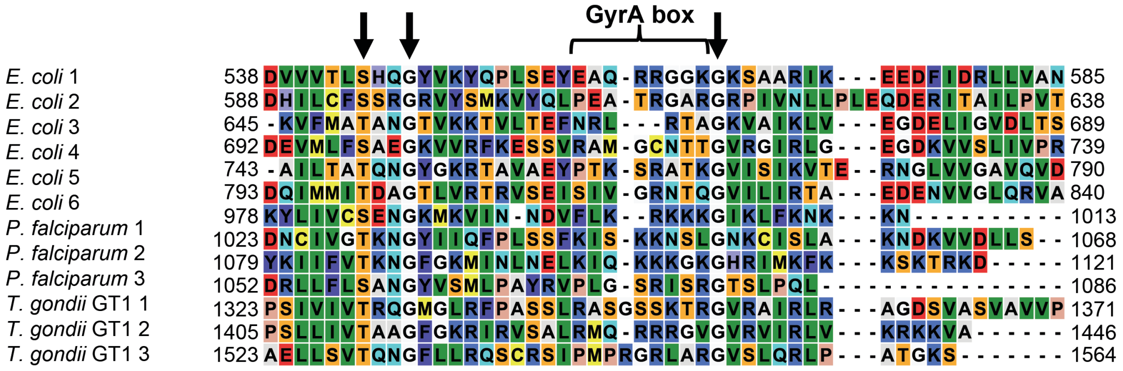

Additional Figure 3: **Asparagine Residues in Pf-gyrase Proteins**

Plasmodium falciparum Gyrase A

MSFKFSIVFVLYLLFLLKFNKRFIFLKSEKITSYINTQIPNYSSSPPFFLKKEAKNNIKK

CYFIKREGYRNIGTKNASNAFVLNFKSNRSTYTDNAFSCTSLCSEKKERKKVQDPYDLKA

KKKEKTYELNDIKNNEKKKDDIVNASNDITNDKLDNINNNINESRKLIKGEYYDVEICEI

LSKSFLSYANFLILNRCLCDYRDGLKTVQRRIIWSMYEINKGIDKKGYKKCARIVGEVIG

KYHPHGDKSVYDALVRLAQKHHNNNLLIKGYGNFGSVEYNAAAMRYTEAKISSFCYDILL

DEINDENVEYIKNFDGNEREPKVLCSKIPLLLINGCSGIAVSILSSIPCHNLIDVANCCI

NFLINENIRDDELFHIIKGPDFSTGGIIISKYDILKNIYNSGKGNFEIRSNVFFEYIKND

KKVITKHINDLSSIENSDIDKLTKKIIIKNLPPNVKPNELIENIINLLNDKKNEHDNILL

RIRDESEKEDMRIVLELKKHSQIEQIHNFLSYLFKYTNMQISYHCNFVCIGYENTYTQFS

LKSFIKLWCNNRIKFIKTNYEIKNKNLQKQLNIIDLYLIIQNKILDIITFFQKNQNIEQI

QLYLKNNFKLNPEQIKYILSIKLQKLINIKNIDFISQRNKIMHQIKLNDEIINNVQNIKN

LIIQELIYIKNKYGIHNLNKQCIIPSTPKYKYSYINSEYHPNFSKNKQIIDTYNTVSTND

NNINHSNYTHDLPNTDNVTKDKNVVETNVHKNIHISSDISNNIKNSDKNSSLTKNQDSDN

MMPYRNTYIDSLDNNMKDIYNNDEVLILITYGGYIKKIKINEKLKNHSNNIIKLSNVKYI

LKENEEKLNENNKRLKFNDLQKGNEQEKYKDNEQKLNNDIGHNINIQNNNNDNNNNNNDN

NNVLLLNEEEYNSYKIKKSILVRNRDKILLTDNYNKAFLLNVYDLHLSSYDSKGTPINQI

IHSSKNITGITKFQENKKYLIVCSENGKMKVINNDVFLKRKKKGIKLFKNKKNIYFSYCN

FNDNCIVGTKNGYIIQFPLSSFKISKKNSLGNKCISLAKNDKVVDLLSYENNEKNLKNYK

IIFVTKNGFGKMINLNELKIQKKKGKGHRIMKFKKSKTRKDNKKDIEKKQINAINNKDKD

KDIPSNNDDNNILHNTKVDEFLGFKLYDMNKQNEKDILIMITDHAVLIRKNMSLLKEKNK

KHSSQIYAKMNKINQLVYFDII

Plasmodium falciparum Gyrase B

MNVRFFKSLSLFIFFVKGVILVVQAGKGNYANIYKKNNINIFNCMKRKYDKKLSSSVVNT

NDKALYESSILKYYYLLDKYKKPNRKKIYGYISNVSRFPLEEERKRKKLEHMNKRNIYPC

YNYDAKDIVILEGLEAVRKRPGMYIGNTDVKGLHQILFEIIDNSVDEYNNFECNTIKVVI

HKDDSVTIEDNGRGIPCDVHEKTKKSALETVLTVLHSGAKFFDDEMDDEIDNNMNNTGNT

NTSEKNNRDNISNNNNNNNNNKRTKKNSKESAQKYKYSSGLHGVGLSVANALSSFMKVKV

FRNNKIYSIELEKGKVTKELTITNCPINKRGTQIHYKPDSSIFKSSTKHNSDLIKNRIHQ

LAYLNEKLTFYFYDERSENKNSIYISKGNKSNEIIKNKKKNTSIDNENEDNKNNTSNYVS

NNTLKDDVSNENENMSDNLVNKNIEITDYNNLDFYNYEIIKHEYGLNEYITNITKNKTNL

FKDNDKIISISCYHKNIYIDLRMKWLSNQYNENIISFVNNVNTTDGGTHVDALKYAVSRC

VNYNIKKNEMIKNFVNIPGEYIREGLTAILSVKMNNPEFEGQTKTKLGSHHLKSILESVI

FEKLSEIFDFEPNLLSSIYFKALQAKLSDEEAKAARDLIRSKNNQYSSTILPGKLVDCIS

DDISRNEIFIVEGDSAAGSAKQARNREIQAILPLKGKILNVEKIKNNKRIFENSELKSLI

TALGLNVNYDNKNLKNNNILSNNKKGKNSKKKSDLKNSRFESTQNNNNILNKKKDILVDN

TLRYGKVIIMTDADVDGEHIRILLLTFLYRFQKEIIENGNVYVACPPLYKITYNKFFDNS

IKDIVTKQFNVTTKQSKFIIHTYSDQELNNLLKLLDKDKIASEQKNMQNKIKNKNMSSNG

EPLSEYNEQSKTNDIFVNEEGSTDSFIDDNVLFNFSKKYEIQRFKGLGEMMADQLWNTTM

DPTVRKLIRVTVNDAIRANDLIFSLMGEDSKLRKNFILENTNSLSE

Additional Figure 4: **Serine Residues in Tg-gyrase Proteins**

Toxoplasma gondii GT1 Gyrase A

MGVFHSPLSSASCFLFFLFVCVFSRCPVHGARLNLPLFSSSTSSQDPSPPALHSFSVSST
LPRSASSHLSSLLRLPFARTAQTTRLLHRQLLSSVSSHIPCFISALPSPSSFSSPSPPSL
PSPSSFSNSGLCSCRRPHACSHSISDGNGYRDGTCCFAGGGLSSSLLPRFIDPSPHERAP
AKKRLPPISVLLRVPPSSLTATASLPSSPPSRLPPFVRLAPLLQSSWCLSPSRASVSSLA
ASEGTLPRTLAKEEGECKERPAPTGSDAKDVALLDTQFVEELEKSFLCYAYSTILSRALP
DVRDGLKPVHRRLIYAMQQLHLHPSGSFRKCARVVGEVLGKFHPHGDQAVYDALVRLAQT
FVARHPLIEGHGNFGSVDGDPPAAMRYTECRLTRFCEDALLKNLDDKVVPFRPNFDANER
EPVVFPASVPLVLIQGSSGIAVGMSTQIPPHNLHEILDATIALIRDPELPDAELLRLVPG
PDFPTGGLLVNSEEIPGAYKEGRGRVLLRGRFHLEGEDDDGDTVPGRDRNAEDEAERDEA
DRHVRNGRKNERRLVITELPYGVNKSTVMQVIAQMISVKFLEGVTSVRDESDWRGIRVVL
VLRRDADAHTILTLLLKHTNLQVYIPMHLIALEKGTKPVRFSLKSMLLSWIAFRFHTLRR
LLAAEEAERKARSHLLEGLLRAVSLMDDVVKTVRESLSADDAKQKLTEPPLGFSPAQAQA
LLRLTLSRLTRLERRQLEEEAKAQKDRLSELASLLAHDREIYELMVEELQAHKTRHKAPR
KTKVLGLRRAQQRARREEDARATTARNSQEAFAETDRHDSRRPDGREKEENPVKEKKETE
ENVQKKDLERGKARTDARHEEGSAGQALEETEGLGGFEEKGRDDRTSTQRKTFAGQDTWR
KDGIVDRQKRLKEEGETETLVSRDVNESSTEAETDASEEDREGDSSEEDQDDARVPFGEI
KNMNKEDFISNQRNVVLLSAPALVRRLPLSVYPTLRRGALGVRSLSRSLASAESPDLERD
EADEAQTRGVKGKSAAVGASSLQGVWSCWTKDRLLFLSANGYVSMLPAYRVPLGSRISRG
TSLPQLLFEGWKKPAALRRLGESAAAAGALAEDAANREKKREEKERTLLREAAQVAAVLA
VPPPTQREKARGVRQASESSGKSRKAKFLVVVTDQGRIAKIKLKRILGRRCETKVKKLLS
LVQGDSIRAAALLEEEVEPLARGRVSGAGKEIKHLRGNGSECNATDEERGARKKDREEED
EETTEKEEEEEDDEEEEDEEDEEEEEEEEAGSRLVEDEDDMGGEGAEGEGTDLKSEEKRDT
NSPSIVIVTRQGMGLRFPASSLRASGSSKTRGVRAIRLRAGDSVASVAVVPPEVKEDEAK
TESRKEGDSDETQSKNESSNEKAEPSLLIVTAAGFGKRIRVSALRMQRRRGVGVRVIRLV
KRKKVAREGDDRGLSDEAVGQESQGFPDEEPDSEERADPRSVSQEKDPQSPETEGDNYVV
AVLALPASDLPNRPKKSKGSDNAELLSVTQNGFLLRQSCRSIPMPRGRLARGVSLQRLPA
TGKSRSSTDLIVAADIVHGASEQQRRRKRRTPED

Toxoplasma gondii GT1 Gyrase B

MKASSERNAGHEKDTKERQLKNTGSCFSPPAGASPPLSSQPSSPETTSSLSSSPRSSSSL
SSSPRSSSSRSSSSRSSSPRSSSSRSSSSRSSSPRSSSSRSSSSRSSSSSSWSSTLCRSS
SSLLSSCPSSPHSLSSSRSSPLSSSSSLSCSRHRISFLHRSVSCRPLPFGRRCAPARQTK
DVFSSSLVFVLFTLSAVSFPAEAVWIPRSCSLLSPHAPSSSDLSAFCVPQSVNRNLLTTL
PAFLPPLKLPKSPLFPPPFSHSLSSSPSLSSLSSSLSSRSSSFDPALLPSSSPPVPSASS
GSTSLLSLPSSGVYASLPSYAFSAFSPSSSPSPSSSPSSSRSFASPSSAYTSDSIRVLEG
LEPVRMRPGMYIGSTDSEGLHHLVWEAIDNAVDEVENGDCRRIVVVLHADRRSVTVADDG
RGIPCDVHPQTGLSALETVFTKLHAGGKFSPASQTGTSSYAFAGGLHGVGSSVVNALSSQ
LRVSVLRDGKLHAISFSRGQVVSPLTVQTSPSLSRGDSAPVGDPRPRETPNEGAKAESEG
DAFAVLPEGNVEAQMRAVEDYVCRFASSSVSPFSRLFQFPSSESQRPPSSGTCLTFEVDR
EIFGDALLDGRKIEQRLRALAYLHPTASFRFFEMPRRRREGMQTEEEASQRLDSHARVES
FPRMSLQVSNENPENLSFRETKRNKQELVRAAESAQPTETEHGQNETEGRTAARTVLFRE
PGGLDSYIRHLSANLTELFSDAPVISINGSHPSSGLLLSVRLFFSSPSSSSASSSSSASS
SSSASSPSSSASSSSSDSSPSSSDSSASASLCSSSSAAASPLSPSSVSSSDFFLSFVNSI
PTPEGGVHVEGIRAALSRAVQRLLRLSDSRSSTFSKSPSTSLSSLSKRDGKRGKGAPGEP
QNSHEKSLNIPGEFLREGLVGVVSLKMREAEFEGQTKKKLGNKKVRGIVEELVTDALLNY
FERHPQNLQKLLQKATAARAAAAAAKAARDFARAKQQSSSLHLTLLPGKLSDCSPTNARE
RLSKELFIVEGESAAGSAKQARDRRTQAILPLRGKILNVEKLGNFARIFENEELKALVAA
LGISVTKAGEVSADLDGLRYSRVIILTDADVDGAHIRSLLLTFFFRLQPELFRQGRIFVA
CPPLFKIAHFPVPHKLLQDATAAFRKRPCSRATSAVPDEARAVHDAHTDEEERGDESDDE
AGRGGGKRLATETYVWSDEEVKRVFEVLRMHRESRKKEQRGPGKRMRGVAADAVLSGGEK
PEHTAREPDRDDGRDGERGGEQDQEEGEERRSSDKTVPGLLEGEADEQLSLPGVTLQRFK
GLGEMQPEQLRRTTMSPVTRILKKISVADAEAAEAAVASLMGDDVDARKKIIFEANRDVF
VEELDV
